# Gender and race disparities in the prevalence of chronic kidney disease among individuals with hypertension in the United States, 2001–2016

**DOI:** 10.3389/fendo.2024.1378631

**Published:** 2024-05-15

**Authors:** Jing Shen, Baoquan Wang, Li Jing, Tiancong Chen, Li Han, Weiwei Dong

**Affiliations:** ^1^ Department of Orthopedics, Shengjing Hospital of China Medical University, Shenyang, China; ^2^ Department of Nursing, Shengjing Hospital of China Medical University, Shenyang, China; ^3^ Department of Neurosurgery, Shengjing Hospital of China Medical University, Shenyang, China

**Keywords:** prevalence, albuminuria, hypertension, chronic kidney disease, NHANES

## Abstract

**Background:**

Chronic kidney disease (CKD) is a common complication among individuals with hypertension. We aimed to identify the prevalence of CKD and the sex and race disparities within the hypertensive population in the United States from 2001–2016.

**Methods:**

A total of 16,148 participants with hypertension were included, representing 561,909,480 individuals from the U.S. population between 2001 and 2016, as documented in the National Health and Nutrition Examination Survey. The prevalence of albuminuria and CKD stage were assessed using survey-weighted general linear regression analysis. Heterogeneity in the CKD stage among the hypertensive population, stratified by sex and race, was identified through survey-weighted logistic regression analysis.

**Results:**

Overall, the prevalence of albuminuria remained stable (p for trend = 0.3196), and changes in the CKD stage were minimal (p for trend > 0.05) from 2001–2016. In the analysis of CKD stage heterogeneity by sex and race, the prevalence of CKD was higher among women than men and higher among individuals of other races combined than non-Hispanic Whites, but the differences were not statistically significant.

**Conclusion:**

The overall CKD stage within the hypertensive population plateaued between 2001 and 2016. Our findings highlight the importance of continuous monitoring and potential refinement of renoprotection strategies in individuals with hypertension to mitigate the persistent burden of CKD and address health disparities among different demographic groups.

## 1 Introduction

Chronic kidney disease (CKD) is a leading cause of the global burden of noncommunicable diseases, contributing substantially to morbidity and mortality and affecting <10% of the worldwide population (1–3). Unhealthy lifestyle choices and comorbidities, including diabetes, hypertension, and poor dietary habits, accelerate the progression of CKD (4, 5). These associations are particularly pronounced within the hypertensive population owing to the interconnected pathophysiological mechanisms linking hypertension and CKD. Specifically, sustained hypertension can exacerbate kidney function deterioration, and CKD can complicate blood pressure management. The complex pathophysiology of hypertension in CKD involves reduced nephron mass, increased sodium retention, activation of the sympathetic nervous system, and the renin-angiotensin-aldosterone system (RAAS) (6–8). Current guidelines recommend a stringent blood pressure target of <130/80 mmHg for individuals with CKD via lifestyle modification and the use of multiple antihypertensive drugs (8, 9). Analyzing the prevalence of CKD and sex and race disparities is crucial for assessing the adequacy of renoprotection strategies in the hypertensive population. Therefore, we investigated changes in CKD trends and assessed CKD heterogeneity by sex and race within the hypertensive population, utilizing the National Health and Nutrition Examination Survey (NHANES) database spanning 2001–2016.

## 2 Materials and methods

### 2.1 Study population

The NHANES is a comprehensive and rigorous database designed to assess the physical and nutritional well-being of noninstitutionalized populations in the United States. The National Center for Health Statistics (NCHS) developed and conducted the NHANES, gathering data on participant demographics, dietary intake, health, and physical examinations through household interviews and mobile examination centers. The NHANES updates its data every two years, making it freely accessible on its website (www.cdc.gov/nchs/nhanes/). Hypertension is defined as an abnormal blood pressure level (systolic blood pressure ≥140 mmHg or diastolic blood pressure ≥90 mmHg), a self-reported physician diagnosis of hypertension, or the use of antihypertensive medications (10, 11). The hypertensive population with complete kidney function data (including urine creatinine, urine albumin, and serum creatinine) consisted of 16,546 individuals from 2001–2016 in the NHANES database. We excluded participants with missing body mass index (BMI) (n=364), serum total cholesterol (n=7), and hemoglobin A1c (n=27) information. Ultimately, 16,148 participants with hypertension were enrolled. All data used in this study were taken from the NHANES publicly available database (https://wwwn.cdc.gov/nchs/nhanes/Default.aspx). The NCHS and Research Ethics Review Committee approved the participation of human individuals in the NHANES study. Written informed consent was provided by each participant.

### 2.2 Measurement of renal function

We utilized the equation developed by the Chronic Kidney Disease Epidemiology Collaboration to calculate the estimated glomerular filtration rate (eGFR) (12). Albuminuria was defined as a urinary albumin-to-creatinine ratio ≥30 mg/g (13). The CKD stage was characterized by an eGFR ≥60 mL/min/1.73 m^2^ with albuminuria or an eGFR <60 mL/min/1.73 m^2^. The severity of CKD was categorized into four stages: no CKD (no albuminuria and eGFR ≥60 mL/min/1.73 m^2^); mild CKD (presence of albuminuria and eGFR ≥60 mL/min/1.73 m^2^); moderate CKD (30 mL/min/1.73 m^2^ ≤ eGFR <60 mL/min/1.73 m^2^); and advanced (eGFR <30 mL/min/1.73 m^2^) (14, 15).

### 2.3 Covariates

Covariates for the multivariable model were selected based on clinical experience and prior research. These covariates included age, sex, race, BMI, serum glucose, hemoglobin A1c, and total cholesterol levels. Diabetes was defined as either a physician-diagnosed condition or the use of hypoglycemic medications or insulin (16). The presence of hyperlipidemia was established when participants had previously received a diagnosis from a physician or were using lipid-lowering medications (17, 18). Smoking status was categorized as never smoked (never smoked or smoked <100 cigarettes in their lifetime), ever smoked (smoked >100 cigarettes in their lifetime), or unknown (19, 20).

### 2.4 Statistical analyses

We adhered to the NHANES analysis guidelines for conducting our statistical analyses, accounting for the complex sample design and employing design variables to determine nationally representative estimates. To enhance statistical accuracy, two-year survey cycles were consolidated into four-year intervals. Continuous variables are reported as survey-weighted means with 95% confidence intervals (95% CIs), whereas categorical variables are presented as survey-weighted percentages with 95% CIs. To assess trends in kidney function changes during the four-year survey periods (2001–2004, 2005–2008, 2009–2012, and 2013–2016), survey-weighted linear regression was used. Survey-weighted logistic regression analysis was performed, and odds ratios (ORs) were calculated to assess cross-sectional heterogeneity in CKD stages by sex and race. We conducted all analyses using R version 3.4.3 (http://www.Rproject.org; The R Foundation) and Empower software (www.empowerstats.com; X&Y Solutions Inc., Boston, MA, USA). Statistical significance was set at p<0.05.

## 3 Results

### 3.1 Baseline characteristics of participants with hypertension from 2001–2016

We enrolled 16,148 participants with hypertension, representing 561,909,480 individuals in the U.S. population. The mean age was 56.29 years, with 49.13% being men. In terms of race, non-Hispanic Whites, non-Hispanic Blacks, Mexican Americans, and other races constituted 71.32%, 13.23%, 5.72%, and 9.73%, respectively. Participants with hypertension exhibited a trend toward poor glycemic control, with the proportion of participants with diabetes increasing from 13.63% to 20.95% between 2001 and 2016 and serum glucose levels rising from 100.62 mg/dL to 109.49 mg/dL. The mean eGFR among participants with hypertension was 84.85 mL/min/1.73 m^2^, and the albuminuria ratio was 15.52%. Detailed information on the basic characteristics and renal function of participants with hypertension from 2001–2016 in the NHANES database is provided in **Table 1**.

**Table 1:** Baseline characteristics of participants with hypertension from 2001–2016.

	All	2001–2004	2005–2008	2009–2012	2013–2016	P-value
Un-weighted sample size	16148	3598	3931	4255	4364	
Weighted sample size	561909480	120044885	136767046	142890665	162206884	
Age,y^a^	56.29 (55.85,56.73)	54.90 (54.13,55.68)	56.04 (54.79,57.30)	57.04 (56.31,57.78)	56.86 (56.22,57.50)	0.0004
gender						0.4558
Male	49.13 (48.24,50.02)	48.02 (45.81,50.24)	49.32 (47.48,51.17)	50.11 (48.48,51.74)	48.93 (47.25,50.62)	
Female	50.87 (49.98,51.76)	51.98 (49.76,54.19)	50.68 (48.83,52.52)	49.89 (48.26,51.52)	51.07 (49.38,52.75)	
Race						0.1614
Mexican American	5.72 (4.70,6.94)	4.62 (2.76,7.63)	5.12 (3.79,6.87)	5.99 (3.98,8.93)	6.80 (4.67,9.79)	
Other races	9.73 (8.79,10.76)	7.49 (5.75,9.70)	7.64 (5.91,9.84)	10.85 (8.65,13.52)	12.16 (10.56,13.98)	
Non-Hispanic White	71.32 (68.67,73.83)	74.96 (69.66,79.61)	74.11 (68.96,78.68)	69.71 (63.56,75.23)	67.67 (62.21,72.69)	
Non-Hispanic Black	13.23 (11.63,15.02)	12.93 (9.89,16.73)	13.12 (9.90,17.19)	13.45 (10.39,17.24)	13.37 (10.30,17.17)	
BMI, kg/m^2 a^	30.70 (30.54,30.87)	30.11 (29.76,30.46)	30.42 (30.12,30.72)	30.91 (30.55,31.26)	31.21 (30.90,31.52)	<0.0001
Serum glucose,mg/dL^a^	106.31 (105.50,107.12)	100.62 (99.38,101.86)	106.06 (104.10,108.01)	107.73 (106.15,109.32)	109.49 (108.00,110.98)	<0.0001
eGFR, mL/min/1.73 m^2 a^	84.85 (84.24,85.45)	85.56 (84.36,86.76)	83.71 (81.99,85.43)	84.91 (83.91,85.92)	85.21 (84.33,86.09)	0.364
Total cholesterol, mg/dL^a^	199.41 (198.32,200.50)	207.84 (205.68,210.00)	200.77 (199.06,202.47)	197.61 (195.40,199.83)	193.61 (191.26,195.96)	<0.0001
Hemoglobin A1c (%)	5.84 (5.82,5.87)	5.72 (5.67,5.76)	5.76 (5.70,5.82)	5.93 (5.89,5.98)	5.93 (5.89,5.97)	<0.0001
Albuminuria						0.1758
No	84.48 (83.69,85.24)	85.26 (83.68,86.71)	83.82 (82.40,85.14)	85.48 (83.74,87.06)	83.58 (81.86,85.17)	
Yes	15.52 (14.76,16.31)	14.74 (13.29,16.32)	16.18 (14.86,17.60)	14.52 (12.94,16.26)	16.42 (14.83,18.14)	
CKD stage						0.4306
No	75.05 (74.08,75.99)	75.64 (73.90,77.29)	74.27 (71.70,76.67)	75.74 (74.09,77.33)	74.66 (72.71,76.51)	
Mild	11.09 (10.46,11.75)	11.12 (9.76,12.64)	11.22 (10.26,12.26)	10.10 (8.74,11.64)	11.83 (10.58,13.19)	
Moderate	12.60 (11.88,13.36)	12.21 (10.84,13.72)	13.21 (11.28,15.41)	12.57 (11.36,13.88)	12.41 (11.19,13.74)	
Advanced	1.26 (1.10,1.45)	1.03 (0.79,1.35)	1.30 (1.01,1.68)	1.59 (1.20,2.09)	1.11 (0.81,1.51)	
Hyperlipemia						<0.0001
No	50.76 (49.59,51.92)	58.09 (55.61,60.53)	54.07 (51.92,56.20)	49.15 (46.85,51.46)	43.95 (41.48,46.45)	
Yes	49.24 (48.08,50.41)	41.91 (39.47,44.39)	45.93 (43.80,48.08)	50.85 (48.54,53.15)	56.05 (53.55,58.52)	
Diabete mellitus						<0.0001
No	82.48 (81.75,83.19)	86.37 (84.86,87.75)	84.11 (82.51,85.59)	81.55 (80.00,83.00)	79.05 (77.44,80.57)	
Yes	17.52 (16.81,18.25)	13.63 (12.25,15.14)	15.89 (14.41,17.49)	18.45 (17.00,20.00)	20.95 (19.43,22.56)	
Smoke status						0.0078
Yes	48.79 (47.67,49.91)	47.81 (45.63,50.01)	47.57 (45.16,49.98)	49.47 (47.61,51.34)	49.95 (47.38,52.52)	
No	50.37 (49.26,51.49)	51.03 (48.89,53.17)	51.36 (49.00,53.72)	49.64 (47.79,51.49)	49.70 (47.10,52.31)	
Unknown	0.83 (0.71,0.98)	1.16 (0.91,1.47)	1.07 (0.74,1.55)	0.88 (0.65,1.19)	0.35 (0.20,0.59)	

CKD, chronic kidney disease; BMI, body mass index; eGFR, estimated glomerular filtration rate.

a Continuous variables presented as survey-weighted mean (95% confidence interval).

Categorical variables presented as survey-weighted percentages (95% confidence interval).

The survey weighting method occasionally produced estimates in decimal numbers. The sum of the numbers may not add up to the heading totals when rounded and added.

### 3.2 Prevalence of albuminuria and CKD status in participants with hypertension from 2001–2016

**Figure 1:**
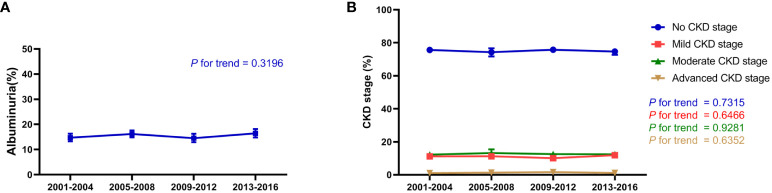
Renal function trend for participants with hypertension in the NHANES (2001–2016). **(A)** Change in albuminuria ratio in participants with hypertension from 2001–2016; **(B)** Change in CKD stage, including no CKD, mild CKD, moderate CKD, and advanced CKD in participants with hypertension from 2001–2016. The estimates are presented as survey-weighted means with 95% confidence intervals. The P for trend was analyzed using survey-weighted linear regression. NHANES, National Health and Nutrition Examination Survey; CKD, chronic kidney disease.

Between 2001 and 2016, the trend in albuminuria exhibited fluctuations but showed no significant change, with albuminuria accounting for 14.74% of the hypertensive population from 2001–2004, 16.18% from 2005–2008, 14.52% from 2009–2012, and 16.42% from 2013–2016 (p for trend 0.3196; **Figure 1A**). The change in prevalence for most CKD stages was minimal, with the prevalence of CKD stages 1–4 remaining relatively stable: no CKD stage (p for trend 0.7315), mild CKD stage (p for trend 0.6466), moderate CKD stage (p for trend 0.9281), and advanced CKD stage (p for trend 0.6352; **Figure 1B**). Overall, the albuminuria and CKD stage did not improve significantly in the hypertensive population over time.

### 3.3 Heterogeneity of CKD stage in participants with hypertension by sex and race

**Figure 2:**
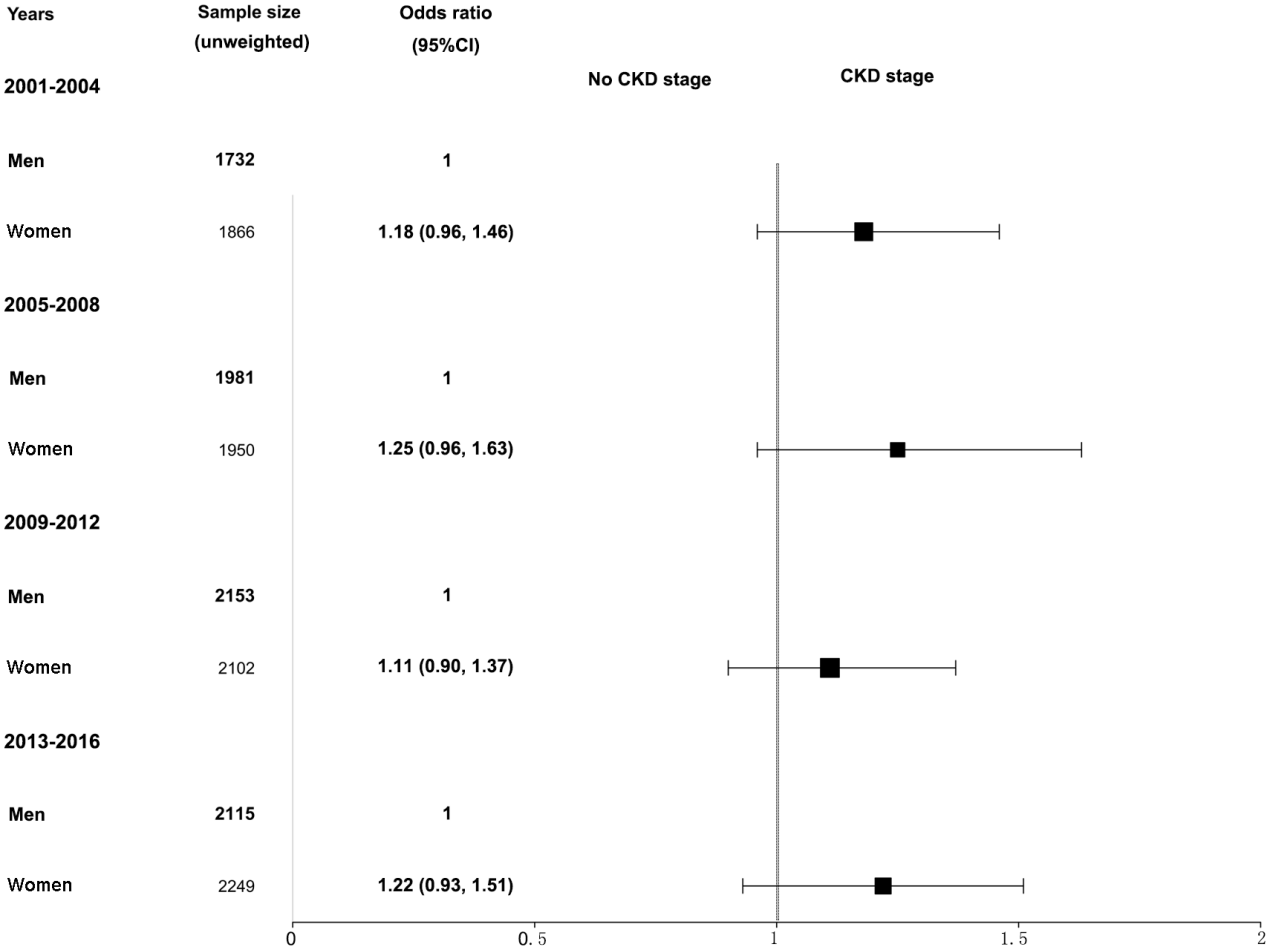
Heterogeneity in the CKD stage in participants with hypertension by sex in the NHANES (2001–2016). The CKD stage was classified as no CKD or existing CKD. An odds ratio >1 indicates a higher risk of CKD. The reference group was male. The analysis results were adjusted for all covariates (age, race, BMI, glucose, total cholesterol, hemoglobin A1c, hyperlipidemia, diabetes mellitus, and smoking status) except for the corresponding subgroup variable, sex. NHANES, National Health and Nutrition Examination Survey; CKD, chronic kidney disease; BMI, body mass index.

**Figure 3:**
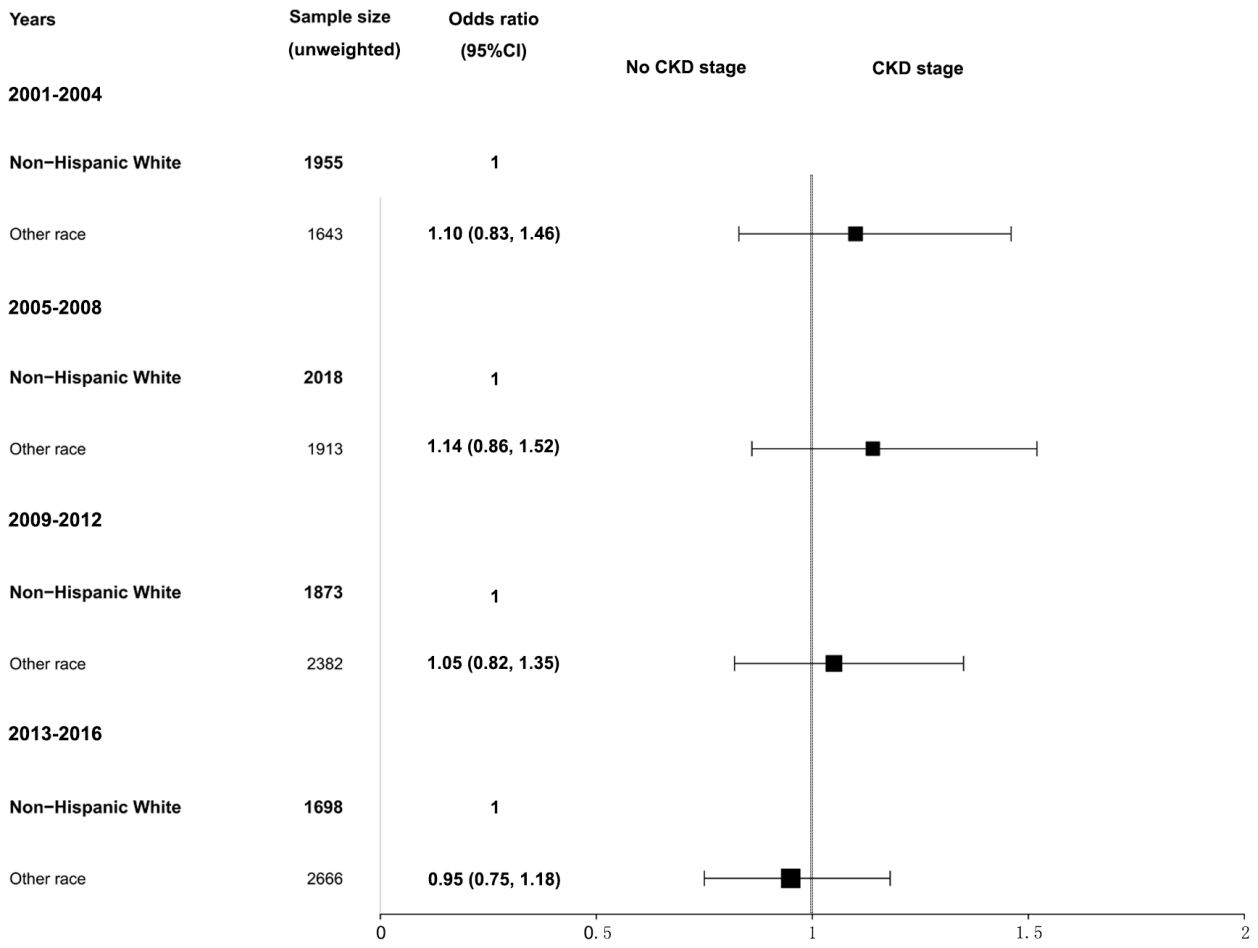
Heterogeneity in the CKD stage in participants with hypertension by race in the NHANES (2001–2016). The CKD stage was classified as no CKD or existing CKD. An odds ratio >1 indicates a higher risk of CKD. The reference group was non-Hispanic White. The analysis results were adjusted for all covariates (age, sex, BMI, glucose, total cholesterol, hemoglobin A1c, hyperlipidemia, diabetes mellitus, and smoking status) except for the corresponding subgroup variable, race. NHANES, National Health and Nutrition Examination Survey; CKD, chronic kidney disease; BMI, body mass index.

Further analysis of CKD stage heterogeneity in participants with hypertension by sex and race from 2001–2016 was conducted. When adjusted for age, race, BMI, serum glucose, hemoglobin A1c, total cholesterol, hyperlipidemia, diabetes mellitus, and smoking, we observed that women had a higher prevalence of CKD than men, but the differences were not statistically significant. The adjusted ORs and 95% CIs for women were 1.18 (0.96, 1.46) in the 2001–2004 survey, 1.25 (0.96, 1.63) in the 2005–2008 survey, 1.11 (0.90, 1.37) in the 2009–2012 survey, and 1.22 (0.93, 1.51) in the 2013–2016 survey (**Figure 2**). Similarly, when adjusted for age, sex, BMI, serum glucose, hemoglobin A1c, total cholesterol, hyperlipidemia, diabetes mellitus, and smoking, participants of other races combined with hypertension had a higher CKD ratio relative to non-Hispanic White participants, and the differences were also not statistically significant. The adjusted ORs and 95% CIs for other races were 1.10 (0.83, 1.46) in the 2001–2004 survey, 1.14 (0.86, 1.52) in the 2005–2008 survey, 1.05 (0.82, 1.35) in the 2009–2012 survey, and 0.95 (0.75, 1.18) in the 2013–2016 survey (**Figure 3**). The disparity in CKD stage between non-Hispanic Whites and other races diminished over the years.

## 4 Discussion

In this study, we revealed a static prevalence of albuminuria and a plateaued prevalence of CKD among participants with hypertension between 2001 and 2016. Furthermore, we observed that women exhibited poorer renoprotection relative to men. We also observed that the prevalence of CKD was higher in other races combined compared with that in the non-Hispanic White population; however, the differences were not statistically significant.

Hypertension is a cause and a factor in the progression of CKD (21, 22). A decline in eGFR is associated with the activation of the RAAS, which exacerbates salt and water retention and heightens salt sensitivity (23). We observed that the CKD stage in the hypertensive population remained relatively stable from 2001–2016. According to the 2017 American College of Cardiology guidelines, all individuals with CKD and hypertension should be treated with a blood pressure target of 130/80 mmHg, regardless of albuminuria (24). Health guidelines from institutions such as the National Institute for Health and Care Research and the UK Renal Association recommend a target of <140/90 mmHg in cases with albuminuria <1g/d and a target of <130/80 mmHg in cases with increased urinary protein leakage. From a therapeutic perspective, angiotensin-converting enzyme inhibitors may be used as first-line agents in patients with hypertension and non-proteinuric CKD (25). Albuminuria is a crucial consideration in hypertension treatment for individuals with CKD, and RAAS blockade appears to offer a blood pressure-independent reduction in albuminuria (26, 27). Implementing this intensive hypertension target in patients with hypertension and CKD would require accurate blood pressure measurement, an understanding of patient preferences and concurrent medical conditions, as well as accurate monitoring of the adverse effects of therapy (28).

The influence of sex on the CKD stage in the hypertensive population remains uncertain. Genetic diversity in the RAAS and *α*-adrenergic receptors may partly explain the CKD heterogeneity between men and women with hypertension (29, 30). Sex hormones also play a role in vascular reactivity (31). Evidence from previous studies has indicated that young men have a higher prevalence of hypertension around the fifth decade of life than women of similar ages until menopause, causing the incidence of hypertension in women to surpass that in men (30, 31). According to an analysis of the 1999–2000 NHANES, White men have a more favorable situation than women regarding hypertension control owing to increasing treatment rates (32). Variations in access to healthcare, health education, and insurance may contribute to racial heterogeneity, resulting in differences in hypertension control. The development of health education programs aimed at preventing and treating hypertension and the expansion of universal health insurance coverage for non-White races may help reduce disparities in CKD stages based on race and sex.

This study had certain limitations. First, variations in urine albumin and blood creatinine testing methods from 2001–2016 might have introduced bias into the results of this study. Second, owing to the limited sample size of non-White races, we categorized races into non-Hispanic Whites and other races when analyzing racial heterogeneity. The lack of detail in racial categorization may miss the opportunity to illustrate the true burden of CKD in hypertensive populations of non-White races. Third, the inclusion of analytic covariates lacked treatment-related variables, such as antihypertensive medication use and type. Fourth, the eGFR value was based on single measurements, potentially excluding some cases of kidney disease. Finally, to obtain more reliable estimates, we combined survey periods into four-year intervals, and it remains uncertain whether the current findings correspond to the 2017–2020 results. Further validation is warranted when the NHANES data is updated.

## 5 Conclusion

Our findings revealed no significant improvement in the CKD stage among the hypertensive population from 2001–2016. The analysis of CKD stage heterogeneity by sex and race indicated a higher prevalence of CKD in women relative to men and other racial groups compared with non-Hispanic White individuals, but the differences were not statistically significant. Further research and greater efforts are needed to refine renoprotection strategies in individuals with hypertension to mitigate the persistent burden of CKD and address health disparities among different demographic groups.

## Data availability statement

Publicly available datasets were analyzed in this study. This data can be found here: https://wwwn.cdc.gov/nchs/nhanes/Default.aspx.

## Author contributions

JS: Data curation, Writing – review & editing, Software. BW: Data curation, Writing – original draft, Software. LJ: Data curation, Writing – original draft, Software. TC: Conceptualization, Data curation, Writing - original draft, Software. LH: Conceptualization, Data curation, Writing – review & editing, Software. WD: Conceptualization, Data curation, Writing – review & editing, Software.
